# PDGFRα/Sca-1 Sorted Mesenchymal Stromal Cells Reduce Liver Injury in Murine Models of Hepatic Ischemia-Reperfusion Injury

**DOI:** 10.1093/stmcls/sxac059

**Published:** 2022-08-24

**Authors:** Andrew Owen, Daniel Patten, Vasanthy Vigneswara, Jon Frampton, Philip N Newsome

**Affiliations:** University of Birmingham, Birmingham, UK; University of Birmingham, Birmingham, UK; University of Birmingham, Birmingham, UK; University of Birmingham, Birmingham, UK; University of Birmingham, Birmingham, UK

**Keywords:** ischemia reperfusion injury, liver transplantation, mesenchymal stromal cells (MSC), liver, stem cells antigen 1 (Sca-1), adult stem cells

## Abstract

Liver transplantation is an effective therapy, but increasing demand for donor organs has led to the use of marginal donor organs with increased complication rates. Mesenchymal stromal cells (MSC) pleiotropically modulate aberrant immune-mediated responses and represent a potential therapy to target the inflammation seen post-transplant with marginal donor livers. To avoid the confounding effects of xenotransplantation seen in studies with human MSC, a PDGFRα/Sca-1 (PaS) sorted MSC population was used which was analogous to human MSC populations (LNGFR^+^Thy-1^+^VCAM-1^Hi^). PaS MSC are a well-described population that demonstrate MSC properties without evidence of clonal mutation during expansion. We demonstrate their anti-inflammatory properties herein through their suppression of T-lymphocyte proliferation in vitro and secretion of anti-inflammatory cytokines (IL-10 and OPG) after stimulation (*P* = .004 and *P* = .003). The MDR2^−/−^ model of biliary injury and hepatic ischemia-reperfusion (HIR) injury models were used to replicate the non-anastomotic biliary complications seen following liver transplantation. Systemic MSC therapy in MDR2^−/−^ mice led to reduced liver injury with an increase in restorative macrophages (5913 ± 333.9 vs 12 597 ± 665.8, *P* = .002, *n* = 7) and a change in lymphocyte ratios (3.55 ± 0.37 vs 2.59 ± 0.139, *P* = .023, *n* = 17), whereas subcutaneous administration of MSC showed no beneficial effect. MSC also reduced cell death in the HIR model assessed by Periodic acid–Schiff (PAS) staining (91.7% ± 2.8 vs 80.1% ± 4.6, *P* = .03). Systemically administered quantum dot-labeled MSC were tracked using single-cell resolution CryoViz imaging which demonstrated their sequestration in the lungs alongside retention/redistribution to injured liver tissue. MSC represent a potential novel therapy in marginal organ transplantation which warrants further study.

## Graphical Abstract

**Figure:**
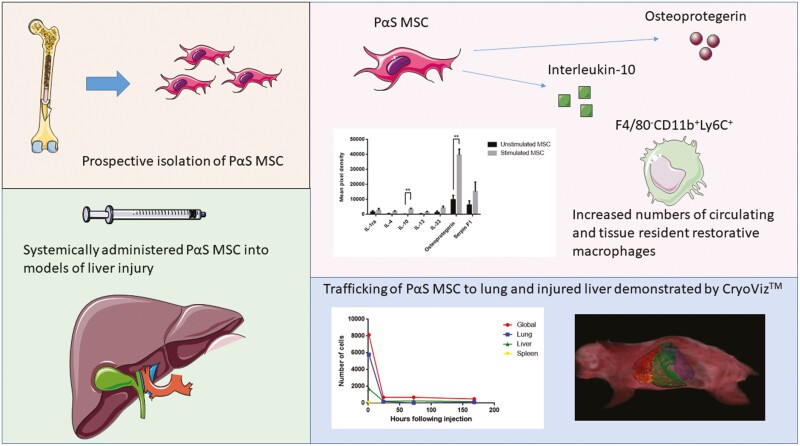
BM PαS MSC secrete OPG and Il-10, traffic to lung, and reduce liver injury

**Significance Statement**

This study demonstrates that purified PαS mesenchymal stromal cells (MSC) are able to reduce immune-mediated injury in models of biliary injury and cell death in ischemia-reperfusion injury. This suggests a role for MSC in marginal organ liver transplantation. Increasing the number of donor organs by improving marginal donor organs will reduce mortality from transplantation and allow for a greater number of patients to receive life-saving surgery. Mechanistic insight into the ability of MSC to polarize both circulating and tissue-resident macrophages adds to existing literature and suggests a role for MSC therapy in other disease processes.

## Introduction

End-stage liver disease is a common end point for prolonged liver injury irrespective of the cause, the curative treatment for which is liver transplantation.^1^ It is estimated that annually there are more than 2 million deaths related to liver disease worldwide and with this an increasing demand for transplantation.^2^ Outcomes are generally good due to rigorous patient and donor selection, but in order to meet the increasing demands on the limited donor pool, there has been increasing use of marginal donor organs with extended donor criteria. This has led to more complications, in particular, biliary complications, which tend to occur in the medium term contributing to increased morbidity and potentially a requirement for re-transplantation.^3^

Marginal organ donation, in particular donation after circulatory death (DCD), leads to an increase in the warm ischemic time to which the donor organ is exposed and thus an increase in damage to the organ.^4^ Warm ischemia is characterized by hepatocyte death whereas cold ischemia leads to loss of sinusoidal endothelium.^5,6^ A variety of mechanisms exist following reperfusion that result in cell death including depletion of cellular ATP and the subsequent failure of ATP-dependent ion channels, free radical generation, and immune-mediated injury following reperfusion.^7-9^ Biliary complications following liver transplantation can be anastomotic (usually smaller and related to the biliary anastomosis) or non-anastomotic (often more diffuse) with the latter more closely associated with DCD transplantation.^3,10^ Non-anastomotic complications are a heterogeneous group of pathologies with a number of possible causes including micro- and macroangiopathy due to cold preservation injury and duration of ischemic time.^11^ Some case series have also implicated immunological injury in biliary complications.^12^ Strategies to reduce biliary complications have been successfully trialed with modifications to surgical techniques to reduce cold ischemic time resulting in a reduction in anastomotic and non-anastomotic complications, respectively.^13-15^ Nevertheless, there remains an incidence of between 1% and 10% of non-anastomotic complications following liver transplantation.^16,17^

There exist a variety of small murine models of immune-mediated liver injury which have been largely accepted as the most practical and relevant human analog. Murine liver transplantation models are complex, challenging, and perhaps do not recapitulate the true conditions seen in a human liver transplantation.^18,19^ Models of hepatic ischemia-reperfusion injury are more robust and represent a simpler and more consistent surgical approach while still providing mechanistic insight into the injury seen following liver transplantation.^20^ Medium-term complications following liver transplantation include non-anastomotic biliary complications due to immune-mediated injury and ischemic cholangiopathy.^21^ The MDR2^−/−^ model develops progressive cytotoxic biliary injury due to genetic defect, leading to ischemic cholangiopathy, lymphocyte infiltration, and the development of biliary fibrosis in keeping with the pathology seen in patients following liver transplantation.^22-24^

Mesenchymal stromal cells (MSC) can either be defined as stem or stromal cells depending on their abilities and phenotype as set out in the International Society for Cell and Gene Therapy (ISCT).^25^ MSC can be used as a novel cell therapy showing increasing promise in liver disease and transplantation,^26,27^ as they have pleiotropic abilities for modulating the aberrant immune responses both in acute and chronic inflammatory conditions.^28^ In particular, MSC can suppress T-lymphocyte proliferation and activation, reduce MHC expression on dendritic cells, and also alter expression of regulatory T cells and macrophages through the secretion of a variety of soluble factors and MSC-derived extracellular vesicles.^27^ However, the MSC literature has been hindered by issues relating to heterogeneity with mixed results seen from a number of clinical and preclinical trials.^29^ Further difficulties in interpreting the results of preclinical trials exist as the majority of studies use human MSC as a cellular therapy in various murine models of inflammatory liver injury. The immune response generated by MSC transplantation has been shown to be present when heat-inactivated human MSC are transplanted into mice suggesting a significant confounding of results in these studies.^30^ These abilities of MSC make them a promising candidate as a therapy to reduce injury and complications of liver transplantation, in particular those seen in marginal donation and DCD transplantation where lymphocyte infiltration is seen early on the following reperfusion and represents a key target for MSC to exert their effect.^31^ Another concern for MSC therapy is the development of instant blood-mediated inflammatory reaction (IMBIR) following intravascular administration, a consequence of tissue factor expression leading to activation of coagulation pathways leading to microvascular occlusion.^32,33^ While an important concern this phenomenon has been largely reported in MSC isolated using plastic adherence and which have undergone significant culture expansion and administration of large doses of MSC.^34^ As MSC can become sequestered in the lungs following systemic administration, alternative routes of administration have been studied to enhance targeting to sites of inflammation.^26,35^ Direct administration to organ systems such as intraportal administration in liver disease has been trialed while subcutaneous administration has shown promise in models of graft versus host disease.^36,37^ MSC administered via the subcutaneous route have been shown to remain at the site of injection and also be equally effective,^36^ a proposed benefit being their persistence rather than rapid clearance when given systemically.^38^

To date, more than 200 clinical trials have been performed using MSC as a therapy in liver disease; however, only a small number have been undertaken in liver transplantation with mixed results.^27^ Rat bone marrow-derived MSC have been shown to reduce ischemic liver injury in a rat model of hepatic ischemia-reperfusion injury by reducing hepatocyte apoptosis; however, the precise mechanism is unclear.^39^ Immune cell recruitment is key in reperfusion injury following transplantation with preclinical data suggesting that bone marrow-derived MSC inhibit neutrophil recruitment via a reduction in CXCR2 expression.^40^ Early clinical trials of MSC therapy in liver transplantation demonstrated safety but not efficacy.^41^

A variety of markers exist for the isolation of murine MSC and are defined by the ISCT.^25^ Murine MSC prospectively isolated using the markers platelet-derived growth factor receptor alpha (PDGFRα) and stem cell antigen-1 (Sca-1), so-called PαS MSC, have been demonstrated to be an ideal homogenous population of MSC capable of tri-lineage differentiation and immunomodulation and represent an ideal cell type to test efficacy in murine models.^42,43^ Since the original description of PαS MSC as a purified population of murine bone marrow-derived MSC capable of tri-lineage differentiation, self-renewal, and plastic adherence,^42^ there have been few studies testing their immunomodulatory efficacy. Notably, when tested in murine models of systemic inflammation, there were no reports of hemo-incompatibility.^42^

In this study, we determined the effects of purified PαS murine MSC in models of liver ischemia-reperfusion injury and biliary injury to investigate their therapeutic potential in marginal organ donation.

## Materials and Methods

### Ethical Statement

All procedures carried out on animals were done in accordance with the Animals (Scientific Procedures) Act 1986, UK. All procedures underwent local ethical review prior to being performed. Procedures were performed under the Home Office project license number 70/7707.

### Animal Husbandry

All animals were housed in accordance with established care protocols at the University of Birmingham in a temperature-controlled sterile animal facility with 12-hour light/dark cycles and free access to food and water. Male MDR2^−/−^ were used at 6-8 weeks of age from an in-house colony. Mice were maintained as homozygous knockout of the mdr2 gene with periodic confirmation of genotype by Transnetyx, USA to ensure no genetic drift.

### Prospective Isolation of PαS MSC

PαS MSC were isolated as originally described by Morikawa et al.^42,43^ Preparation of cell suspensions and antibody staining was carried out as described. Cell sorting was performed using a MoFlo XDP (Beckman Coulter).

### Cell Culture and Growth Factor Priming of Cultured PαS MSC

Prospectively isolated PαS MSC cultured in standard media consisting of αMEM (α-modified Minimum Essential Media, Gibco UK) containing 10% heat-inactivated fetal bovine serum (FBS, Gibco UK) and 1× penicillin-streptomycin-glutamine (PSG, Gibco UK) under standard conditions (37°C, 5% CO_2_) in a tissue culture incubator. Cell passage was undertaken when 90% confluence was achieved using TrypLE Express (Gibco UK).

### MDR2^−/−^ Murine Model of Liver Injury

The MDR2^−/−^ mouse model with Friend virus B-type/N (FVB) genetic background was used as described by Fickert et al.^24^ Randomly selected male MDR2^−/−^ mice aged 6-8 weeks were injected with either phosphate-buffered saline (PBS) or PaS MSC at passage 4 suspended in PBS. This time point was chosen based on the earlier work by Fickert et al which demonstrated peak immune cell infiltration with minimal fibrosis development at this time point.^22-24^ Either 1 × 10^5^ or 2 × 10^5^ PaS MSC were used and diluted into a volume of 100 µL for intravenous injection via the tail vein and 200 µL for subcutaneous injection. MSC were removed from culture flasks by trypsinization with TrypLE Express (Gibco UK). MSC were washed and resuspended in PBS for counting followed by dilution to the required volume and concentration. Cells were used fresh and never frozen in order to preserve function^44,45^ and no anti-coagulation was used. Immediately prior to injection, MSC were mixed by gentle pipetting and filtered through a sterile 50 µm filter (Partec, DE) and then drawn up into a 29-g insulin needle (Terumo, USA). Two weeks after the MSC administration, blood samples were collected via cardiac puncture under terminal isofluorane anesthesia.

### Hepatic Ischemia-Reperfusion Model

The hepatic ischemia-reperfusion injury model was developed in-house based on the published literature.^20^ Male C57BL/6 mice were purchased from Charles River Laboratories and used at 8-10 weeks of age. One hour prior to the induction of ischemia either PBS control or 1 × 10^6^ PαS MSC were injected via the intraperitoneal route. Cell preparation was carried out following the same procedures as for the MDR2^−/−^ model. Ischemia was induced for 1 hour using an atraumatic clamp and following recovery, the experiment was run for 24 hours, and afterwards tissue samples were collected for further analysis.

### Histopathology and Immunohistochemistry

For morphological analyses, mouse liver tissue specimens were fixed in 4% paraformaldehyde, embedded in paraffin, and cut into 4 µm sections for fixing onto X-tra Adhesive microscope slides (Leica, UK). Tissues slides were deparaffinized according to standard procedures prior to staining with freshly made hematoxylin and eosin (H&E). Finally, slides were mounted using DPX and imaging with conventional light microscopy (Carl Zeiss, UK).

Immunohistochemistry (IHC) analyses were performed using 3,3ʹ-diaminobenzidine (DAB) staining standard protocol. Briefly, paraffin-embedded mouse liver sections were deparaffinized and rehydrated. High-temperature antigen retrieval was performed using antigen-unmasking solution (Tris-EDTA, H-3301; Vector Laboratories, UK) for 15 minutes. Endogenous peroxidase activity was blocked with pre-diluted peroxidase-blocking solution (S2023; Dako, UK) for 15 minutes with gentle rocking. Slides were washed with wash buffer containing Tris-buffered saline (TBS; T9141, TaKaRa Bio Inc, Europe) and 0.1% Tween-20 (P2287; Sigma-Aldrich, UK) and blocked with casein solution (SP-5020; Vector Laboratories, UK) for 15 minutes at room temperature. The liver sections were then incubated with primary antibodies optimally diluted in TBS as follows: rat monoclonal anti-mouse CD45 (1:200; eBioscience, 14-0451); rat monoclonal anti-mouse F4/80 (1:200; eBioscience, 14-4801); anti-mouse CK19 (1:500, TA300867, Origene) for 1 hour with gentle rocking at room temperature. Subsequently, slides were washed and incubated with either anti-rat IgG or anti-rabbit IgG ImmPRESS HRP (horseradish peroxidase) conjugated secondary antibody (MP-7444 and MP-7451; Vector Laboratories, UK) for 30 hours with gentle rocking at room temperature. Staining was developed and visualized in brown chromogen using ImmPACT DAB Peroxidase Substrate Kit (SK-4105; Vector Laboratories, UK).

### Splenocyte Isolation

The spleens from OT1 mice were dissociated into C10 media using a 70 µm filter, and red cell lysis was performed. Cells were stained with CellTrace Violet Cell Proliferation Kit and then 1 × 10^5^ or 2 × 10^5^ in 200 µL of C10 media seeded onto a 96-well plate. Stimulation was achieved with OVA_257-264_ peptide and after 24 hours graded doses of PαS MSC were added. After 72 hours, cells were removed, stained, and analyzed by flow cytometry using a CyAn ADP Flow Cytometer. Analysis was performed offline using FlowJo version X.0.7 (TreeStar, USA).

### Protein Array

Protein profiling of cell culture supernatant was undertaken using a Proteome Profiler (R&D Systems, USA). PαS MSC were cultured to passage 5, and some flasks were stimulated with 20 ng/mL of murine IFN-γ (PeproTech, UK) and 20 ng/mL murine TNF-α (PeproTech, UK). Protein profiling was carried out following the manufacturer’s instructions. Protein arrays contain internal positive and negative controls and undergo normalization across each blot allowing semi-quantitative representation of expression data. Blots were processed using x-ray imaging, and images were scanned and analyzed with ImageJ.

### Cell Tracking by Cryo-imaging

PaS MSC were cultured as previously described. Cells were removed from flasks by incubation with TrypLE Express and washed. Labeling was carried out using Qtracker 605 Cell Labelling Kit (Thermo Fisher Scientific, USA). Labeling solution was prepared according to the manufacturer’s instructions. For every 1 × 10^6^ PaS MSC 1.5 mL of labeling solution and 0.2 mL of standard medium were combined. Cells were incubated at 37°C for 60 minutes in a standard culture incubator in the dark. Following incubation cells were washed twice with standard medium at 280 *g*. Cells were either resuspended in PBS for staining, FACS, or for injection into mice, or were resuspended in standard medium and placed in flasks for further culture. In a subset of experiments, live/dead staining was performed prior to cell labeling as previously described.

Cryo-imaging was undertaken on whole mice and individual organs. Organs were carefully removed from sacrificed mice and injected ex vivo with QDot_605_-labeled PaS MSC. Organs were then placed in foil baths and immersed in optimal cutting temperature compound (OCT; Sakura Finetek, USA). Organs were then frozen on dry ice and stored at −80°C prior to shipping to BioInVision (USA) for analysis. MDR2^−/−^ mice were injected with QDot_605_-labeled PaS MSC and culled at different time points using a CO_2_ chamber. Mice were placed in foil baths and completely submerged in OCT. Mice were then frozen on dry ice and stored at −80°C prior to shipping to BioInVision. Samples were sectioned and imaged on the CryoViz instrument, and quantification and image generation were undertaken by the technical staff at BioInVision.

### Analysis of Liver Tissue

#### Liver Digest

Liver lobes were weighed and then placed into gentleMACS C Tubes (Miltenyi, DE) with 10 mL of Roswell Park Memorial Institute media (RPMI; Gibco, UK). Following processing samples were passed through a 70 µm mesh and washed. Samples were centrifuged at 50*g* for 10 minutes. Supernatant was collected and washed three times. After passing through a 50 µm filter 7 mL of suspension was carefully added to 7 mL of Lympholyte Mouse Cell Separation Media (Cedarlane, USA). Samples were then centrifuged at 280 *g* for 30 minutes with no brake. The interphase layer of lymphocytes was then removed and washed in FACS buffer before live dead staining using a live/dead dye (1 L/mL; eBioscience, UK). Following washing cell surface staining was undertaken for CD3-BV510 (5 µL; Biolegend, UK), CD3-PE (5 µg/mL; eBioscience, UK), CD3-APC (2.5 µg/mL; eBioscience, UK), CD3-PE-Cy7 (10 µg/mL; eBioscience, UK), CD4-BV421 (5 µL; Biolegend, UK), CD4-FITC (2.5 µg/mL; eBioscience, UK), CD4-PerCP (1:100; eBioscience, UK), CD8-PE-Cy7 (5 µg/mL; eBioscience, UK), CD8-APC (1.25 µg/mL; eBioscience, UK), CD8-APC-Cy7 (1:100; BD Biosciences, UK), CD19-APC (1:100; BD Biosciences, UK), CD25-PE (1.25 µg/mL; eBioscience, UK), CD25-APC (1.25 µg/mL; eBioscience, UK), CD45-PE (1 µL/mL; eBioscience, UK), CD69-FITC (5 µg/mL; eBioscience, UK), CD45-Pacific Blue (2.5 µg/mL; eBioscience, UK), F4/80-FITC (5 µg/mL; eBioscience, UK). Samples were run on a CyAn ADP Flow Cytometer (Beckman, UK) and analyzed using FlowJo (TreeStar, USA) with cell counts normalized to liver weight.

## Results

### Stimulated PαS MSC Suppress Lymphocytes and Secrete IL-10 and Osteoprotegerin

**Figure 1.**
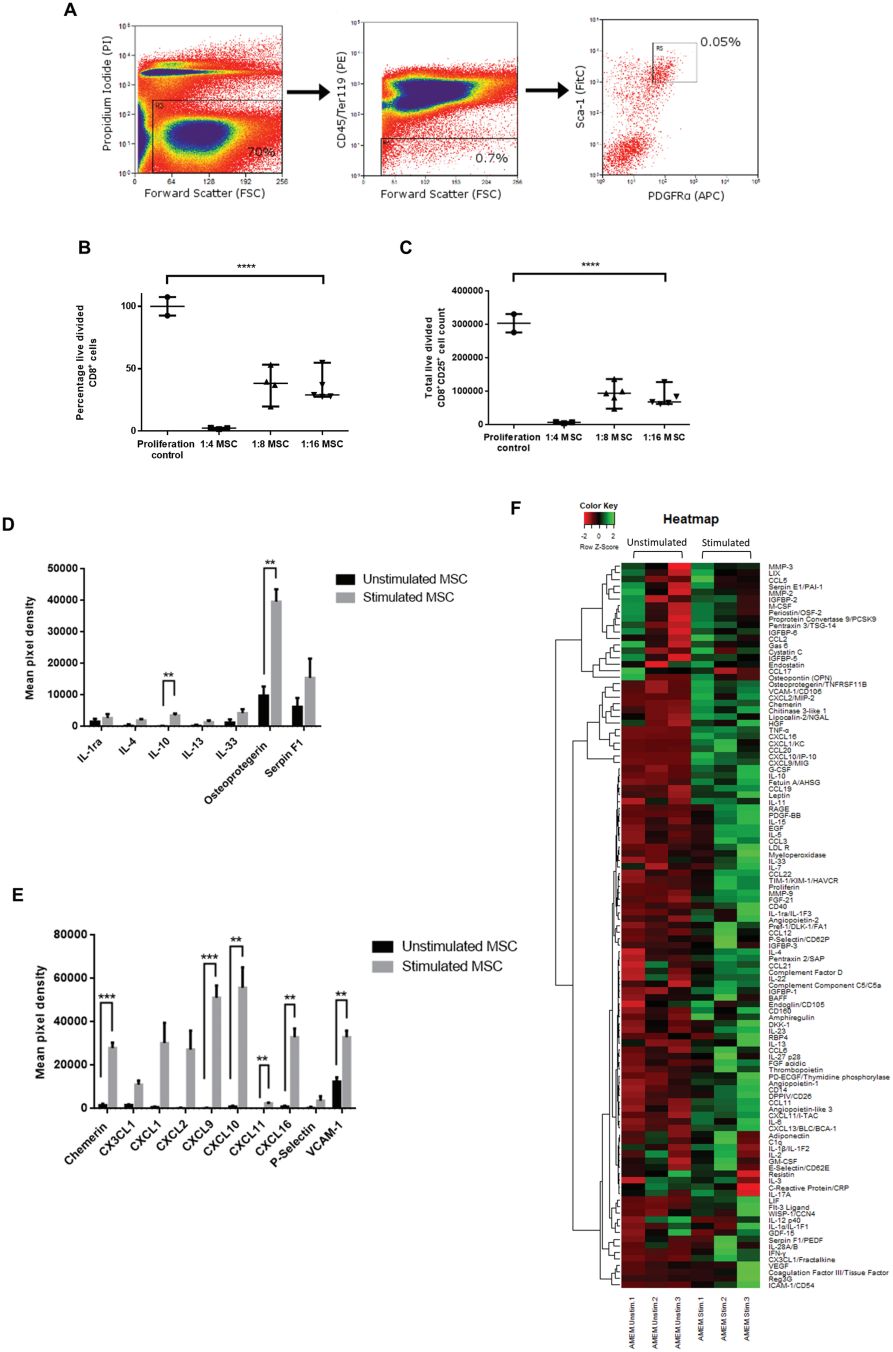
Isolation and characterization of the secretome and immunosuppressive action of PαS MSC. (A) The flow cytometric gating strategy for isolating PαS MSC is shown. Viability was determined using propidium iodide followed by negative selection for cells without CD45 or Ter119 expression. Finally, cells were sorted on PDGFRα and Sca-1. (B, C) In an in vitro bulk splenocytes reaction PαS MSC were able to reduce the numbers of CD8^+^ lymphocytes and C8^+^CD25^+^ activated lymphocytes. Mixed immune cells from OT1 mice were stimulated with OVA_257-264_ peptide in combination with IL-2 and after 24 hours graded numbers of PαS MSC were added. The total number of live divided CD8^+^ lymphocytes (*P* < .0001) and C8^+^CD25^+^ activated lymphocytes (*P* < .0001) were significantly reduced (*n* = 15). (D, E) Following in vitro stimulation with 20 ng/mL each of IFN-γ and TNF-α the secretome of PαS MSC was assessed with a Proteome Profiler demonstrating significant increases in the anti-inflammatory cytokines IL-10 and osteoprotegerin (*P* = .00453 and *P* = .00363), and significant increases in the adhesion molecules Chemerin (*P* = .000533), CXCL9 (*P* = .000780), CXCL10 (*P* = .00432), CXCL11 (*P* = .00238), CXCL16 (*P* = .00132) and VCAM-1 (*P* = .00497). Multiple *t*-tests with Bonferroni’s corrections were undertaken (*n* = 6). (F) A heatmap demonstrates changes in all the secreted cytokines assessed using a Proteome Profiler.

Purified PαS MSC were isolated from the long bones of male C57BL/6 mice as originally described by Morikawa,^42^ yielding approximately 10 000 cells per mouse used (Fig. 1A). Our laboratory has extensively characterized PαS MSC and demonstrated surface marker expression in keeping with the minimal criteria described by the ISCT (Supplementary Fig. 1A). Following in vitro culture with OT1 lymphocytes stimulated with OVA_257-264_ peptide in a mixed immune cell culture there was a suppression of both CD8^+^ lymphocyte proliferation (*P* < .0001, *n* = 14) and activation (CD8^+^CD25^+^ lymphocytes; *P* < .0001, *n* = 15) when compared with untreated controls (Fig. 1B, 1C). To assess the secretome of PαS MSC following exposure to conditions of inflammation, cells were stimulated in vitro with TNF-α and IFN-γ resulting in significant increases in anti-inflammatory cytokines IL-10 (*P* = .004), osteoprotegerin (*P* = .003) (Fig. 1D), and adhesion molecules and chemokine receptors (Fig. 1E), including Chemerin (*P* = .000533), CXCL9 (*P* = .000780), CXCL10 (*P* = .00432), CXCL11 (*P* = .00238), CXCL16 (*P* = .00132) and VCAM-1 (*P* = .00497), with all molecules tested demonstrated on a heatmap (Fig. 1F).

### Systemic Administration of PαS MSC Reduces Liver Injury in MDR2^−/−^ Mice by Polarizing Macrophages to a Restorative Phenotype

**Figure 2.**
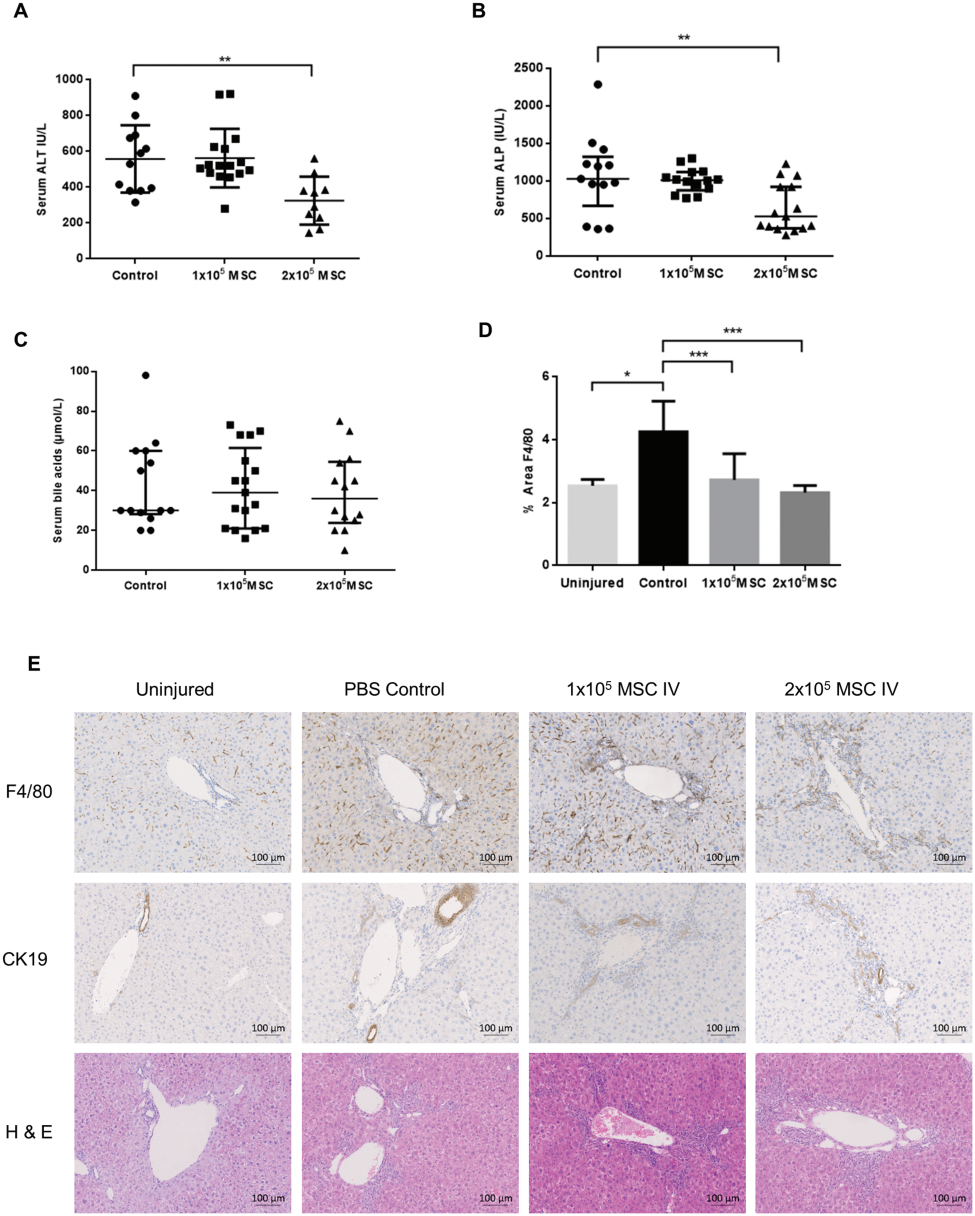
PαS MSC reduce liver injury in the MDR2^−/−^ mouse model. PαS MSC reduce liver injury in the MDR2^−/−^ mouse model a significant reduction in (A) ALT 325.7 IU/L ± 191.7 vs 562 IU/L ± 87.3, *P* = .002, *n* = 38) and (B) ALP 1067 IU/L ± 319.1 vs 635.1 IU/L ± 178.7, *P* = .0055, *n* = 42) but no changes in (C) serum bile acids (39.07 mmol/L ± 11.27 vs 42.93 mmol/L ± 12.85, *P* = .974, *n* = 45) 2 weeks after treatment with 2 × 10^5^ IV PαS MSC compared with PBS controls. (D) Quantification of F4/80 staining in MDR2^−/−^ livers was significantly increased when compared with wild-type mice (4.257 ± 0.896 vs 2.537 ± 0.514, *P* = .0185) and 2 weeks following PαS MSC treatment, there was a significant dose-dependent reduction in F4/80 (4.257 ± 0.896 vs 2.725 ± 0.46, *P* = .0011, 4.257 ± 0.896 vs 2.322 ± 0.235, *P* = .0006, *n* = 31). (E) Representative immunohistochemical images demonstrating periportal localization of F4/80 staining with MSC treatment and biliary tract CK19 staining and representative H&E staining.

**Figure 3.**
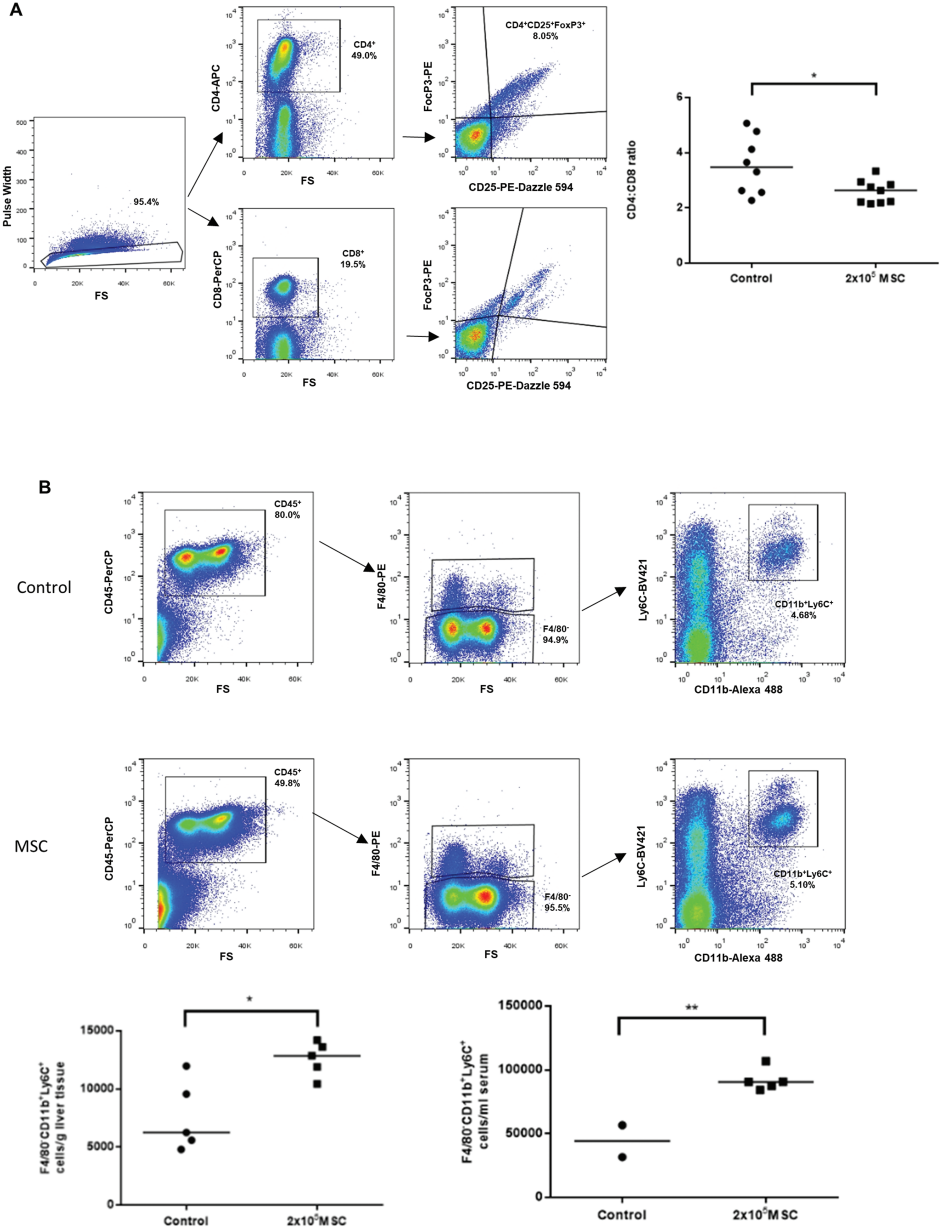
PαS MSC polarize macrophages to a restorative phenotype in the MDR2^−/−^ mouse model. (A) Gating strategy for T regulatory cells in MDR2^−/−^ mouse livers 2 weeks following systemic PαS MSC treatment showed no significant changes; however, there was a significant reduction in the CD4:CD8 ratio (3.55 ± 0.37 vs 2.59 ± 0.139, *P* = .023, *n* = 17). (B) Gating strategy for restorative macrophage populations showed a significant increase in restorative macrophages in both liver (5913 ± 333.9 vs 12 597 ± 665.8, *P* = .002, *n* = 7) and serum (44 104 ± 12 506 vs 90 427 ± 3905, *P* = .0036, *n* = 7) of MDR2^−/−^ mice following treatment with PαS MSC.

The effects of systemically administered MSC were studied in the MDR2^−/−^ model of biliary injury.^46^ PαS MSC were administered to mice at 6-8 weeks of age when immune-mediated injury peaks.^22^ Reductions in alanine transaminase (ALT) (*P* = .002) and alkaline phosphatase (ALP) (*P* = .0055) were seen with higher doses of MSC but there were no changes in levels of serum bile acids (*P* = .974) following systemic administration (Fig. 2A-2C). Immunohistochemical analysis demonstrated a significant reduction in F4/80^+^ cells (*P* = .006) following MSC therapy (Fig. 2D, 2E). Flow cytometric analysis (Fig. 3A, 3B) showed no significant changes in CD4^+^ or CD8^+^ lymphocyte numbers; however, there was a reduction in the CD4^+^/CD8^+^ ratio (*P* = .023) in keeping with a regulatory rather than cytotoxic lymphocyte phenotype.^47,48^ Macrophage populations took on a restorative phenotype (F4/80^−^CD11b^+^Ly6C^+^) following systemic MSC therapy in both liver tissue (*P* = .002) and serum (*P* = .0036).

### Systemically Administered PαS MSC Are Cleared from All Organs Rapidly But May Redistribute to the Livers of MDR2^−/−^ Mice Following Systemic Administration

**Figure 4.**
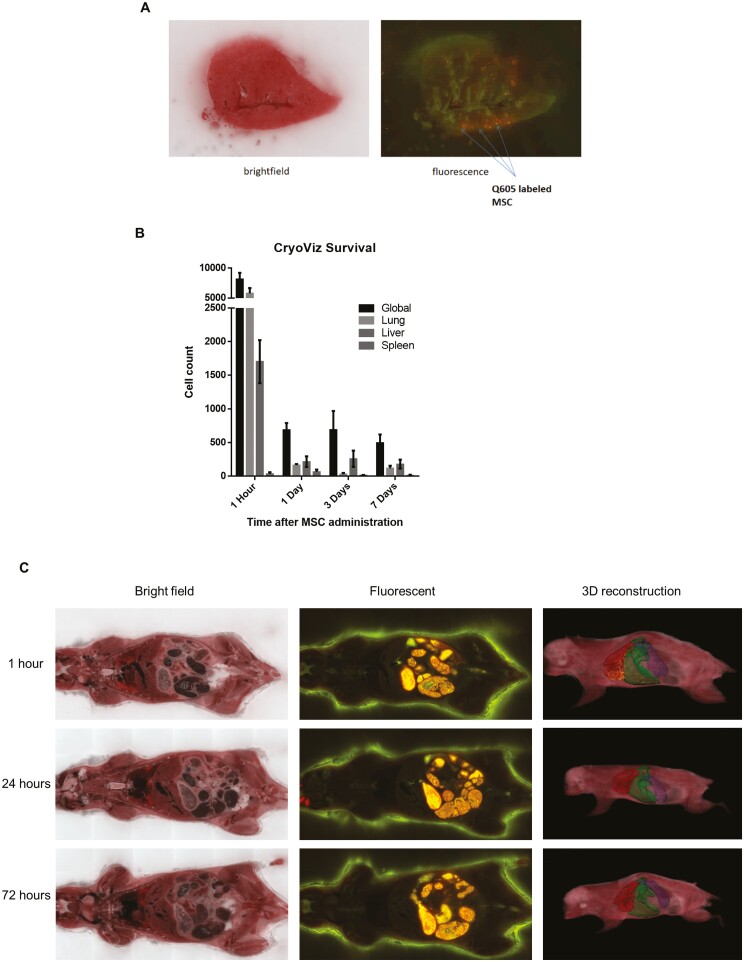
Systemically administered PαS MSC home to the lungs and redistribute to the liver in MDR2^−/−^ mice. (A) Representative bright field and fluorescent images of 8-week-old MDR2^−/−^ mouse lung tissue directly injected with 1 × 10^5^ PαS MSC labeled with QDot_605_ used for calibration of the CryoViz system. (B) Following IV injection with PαS MSC 8-week-old male MDR2^−/−^ mice were culled, frozen in OCT, and analyzed using CryoViz at different time points (*n* = 12). There was a rapid decline in all organs in the first 24 hours with the greatest numbers seen in the lungs. There was a slower continuous decline over the remaining 7 days; however, the rate of decline was lower in the liver than in the other organs. Bar graph demonstrating cell counts in all organs over the time course of the experiment represented as mean and SEM. (C) Representative bright field, fluorescent, and 3D reconstructed images from CryoViz analysis.

We next investigated the distribution of systemically administered MSC in the MDR2^−/−^ model by using the novel cryo-imaging technique CryoViz in collaboration with BioInVision. MSC labeled with QDot_605_ were used to calibrate the CryoViz system in single organs (Fig. 4A). Once calibrated single-cell tracking of systemically administered MSC was performed and showed that the majority of cells were located in the lungs at 1 hour (Table 1, Fig. 4B, 4C) in keeping with published studies of more heterogeneous populations of murine MSC.^49^ While cells were rapidly cleared from all studied tissues over the course of the experiment, the percentage of cells present in the liver increased over the 7-day study period which could indicate retention or redistribution from other tissues.

**Table 1.** The rate of decline in different organs of PaS MSC when administered to MDR2^−/−^ mice.

Time point (hours)	Location		
	Lung (%)	Liver (%)	Spleen (%)
1	71.1	20.9	0.4
24	23.5	31.5	9.5
72	4.3	37.6	1.9
168	23.7	26.4	2.3

Male 8-week-old MDR2^−/−^ mice were injected into the tail vein with 1 × 10^5^ passage 4 QDot_605_-labeled PaS MSC. The majority of cells appear to stay in the lungs during the first 1 hour with 71.1% of cells being found in the lungs compared with just 20.9% of cells being found in the liver. However, at 24 hours only 23.5% of cells are found in the lungs, with 31.5% being found in the liver. This trend appears to be maintained over the 7-day period with a greater percentage of cells being found in the liver when compared with the lungs (*n* = 12).

### Subcutaneous Administration of PαS MSC in the MDR2^−/−^ Model

**Figure 5.**
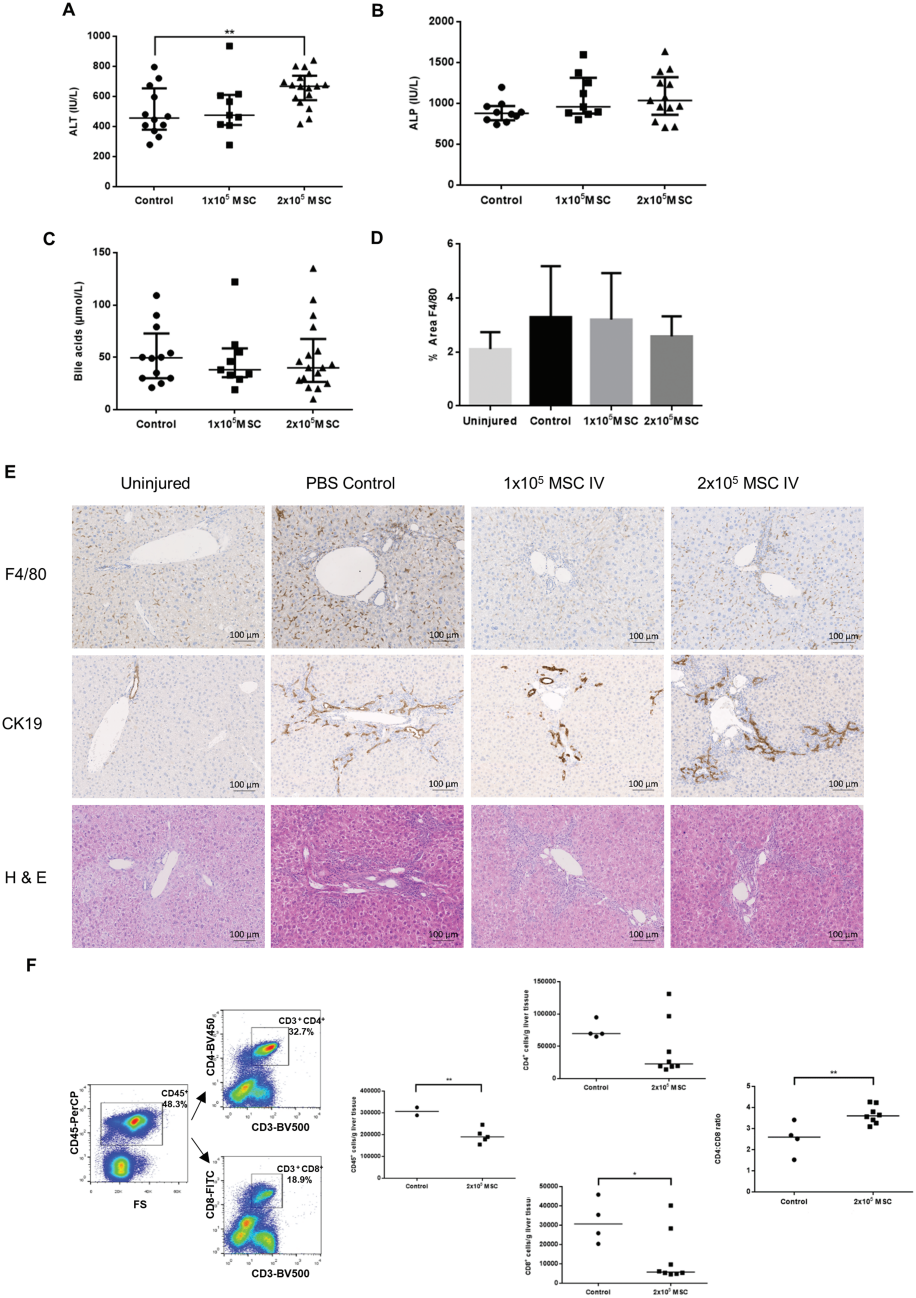
Subcutaneously administered PαS MSC reduce lymphocyte numbers but do not reduce liver injury in MDR2^−/−^ mice. Male MDR2^−/−^ mice aged 6-8 weeks treated with subcutaneous PαS MSC showed (A) a significant increase in serum ALT (652.5 ± 28.8 vs 497.8 ± 46.9, *P* = .0062, *n* = 38) but no significant difference in (B) serum ALP (895.9 ± 94.6 vs 1085 ± 174, *P* = .0676, *n* = 32) or (C) serum bile acids (51.83 ± 17.5 vs 50.29 ± 17.28, *P* = .897, *n* = 28) after 2 weeks. (D) Immunohistochemical analysis of F4/80 in mouse livers showed no significant changes after PαS MSC treatment (3.296 ± 0.598 vs 2.583 ± 0.187, *P* = .186). (E) Representative images of immunohistochemical staining demonstrate scattered F4/80 expression throughout the liver and CK19 in the biliary region. (F) Flow cytometric analysis of MDR2^−/−^ mouse livers demonstrated a significant reduction in CD45^+^ (306 551 cells/g ± 18 165 vs 194 496. cells/g ± 15 135, *P* = .009, *n* = 7) and CD8^+^ lymphocytes (31 855 cells/g ± 5589 vs 13 079 cells/g ± 4787, *P* = .0385, *n* = 12) and a significant increase in the CD4:CD8 ratio (*P* = .0078) following PαS MSC treatment.

MSC have been shown to exert local and systemic effects with the subcutaneous route of administration an attractive therapeutic option. We sought to characterize the effects of subcutaneously administered MSC in the MDR2^−/−^ model of liver injury. When administered via the subcutaneous route there was a significant increase in liver injury characterized by a rise in ALT (Fig. 5A, *P* = .006). No changes were seen in serum ALP and bile acids (Fig. 5B, 5C), and semi-quantitative IHC showed no changes in F4/80 expression (Fig. 5D). IHC demonstrated a scattering of F4/80 cells around the liver tissue with CK19 cells concentrated around the biliary tree (Fig. 5E). In contrast to the findings following systemic administration, the subcutaneous route led to a reduction in CD45^+^ (*P* = .009) and CD8^+^ (*P* = .04) cells with no significant changes in CD4^+^ cells; however, this resulted in a significant increase in the CD4:CD8 ratio (*P* = .008) suggesting a shift toward a cytotoxic lymphocyte response (Fig. 5F).

### PαS MSC Reduce Ischemic Tissue But Not Markers of Liver Injury in Hepatic Ischemia-Reperfusion Injury

**Figure 6.**
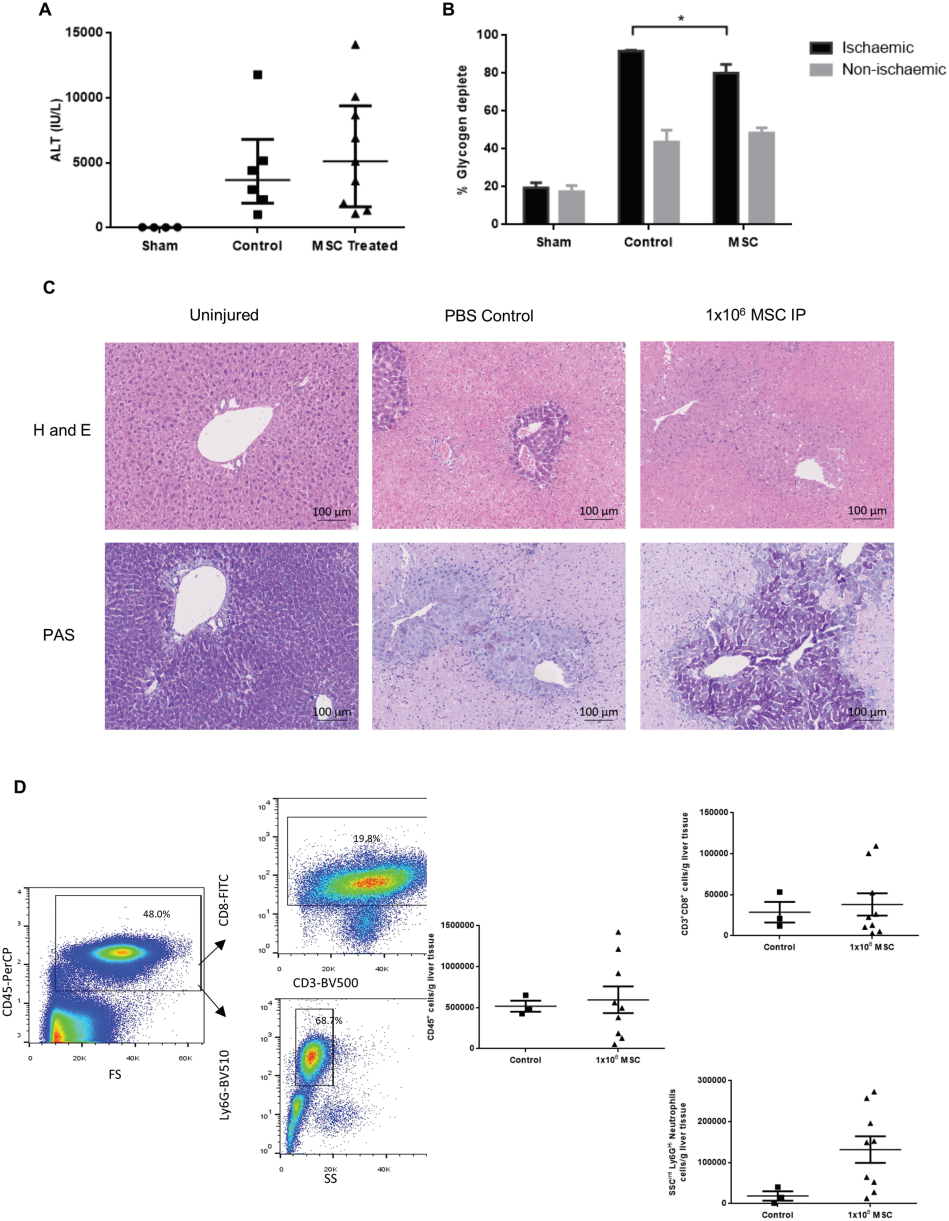
PαS MSC reduce the amount of damaged cells but not the ALT in hepatic ischemia-reperfusion injury. Male C57BL/6 mice aged 8 weeks treated with 1 × 10^6^ intraperitoneal PαS MSC showed (A) no change in serum ALT (4585 IU/L ± 1567 vs 5877 IU/L ± 1491, *P* = .57, *n* = 12) but a significant reduction in (B) glycogen depletion in the ischemic lobes 91.7% ± 2.8 vs 80.1% ± 4.6, *P* = .03), whereas the non-ischemic lobes showed no significant difference (43.6% ± 6.4 vs 48.4% ± 2.7) when assessed with PAS staining (*n* = 12). (C) Representative H&E and PAS staining demonstrate islands of preserved cells around the vasculature with widespread loss of hepatocytes across the liver sections. (D) Flow cytometric analysis of IR mouse livers demonstrated no significant changes in CD45^+^ (517 939 cells/g ± 67 547 vs 596 245 cells/g ± 162 957, *P* = .79, *n* = 11), CD8^+^ lymphocytes (28 744 cells/g ± 12 570 vs 38 185 cells/g ± 13 582, *P* = .72, *n* = 11) or neutrophils (18 753 cells/g ± 11 449 vs 131 812 cells/g ± 32 555, *P* = .08, *n* = 11) following PαS MSC treatment.

We next sought to assess the effects of PαS MSC in a model of hepatic ischemia-reperfusion injury. MSC were delivered 1 hour prior to surgery via intraperitoneal injection in order to allow adequate time for engraftment of MSC to liver tissue while avoiding the added insult of systemic or portal administration. Twenty-four hours after recovery from 60 minutes of 70% clamping there was no significant reduction in ALT (Fig. 6A) and no changes in serum or liver infiltrating immune cells (Fig. 6D). There was, however, a significant reduction in glycogen depletion in hepatocytes (Fig. 6B), a marker of cell death indicating significantly less cell death in the lobes that underwent ischemia but not in those that were spared (*P* = .03) by the 70% clamping strategy (Fig. 6B, 6C).

## Discussion

In this study, we have shown that systemically administered PαS MSC are able to reduce liver injury with a concomitant increase in hepatic and circulating restorative macrophages, while subcutaneously administered PαS MSC confer no benefit and may in fact lead to harm.

Ischemia-reperfusion injury has been a major problem for organ transplantation since the first transplant operations. With a greater understanding of the pathophysiology and optimization of donation pathways, the focus has now shifted to the immune system and its role in organ damage. With the use of DCD organs comes a change in the types of complications seen with the increased presence of the non-anastomotic biliary complications. In this study, we have suggested a potential role for MSC in reducing these complications from transplantation. By using the MDR2^−/−^ mouse model of biliary injury we have closely recapitulated the pathology seen in non-anastomotic biliary complications of DCD transplantation including microangiopathic injury.^22-24^

We have shown that PαS MSC are able to secrete IL-10 and OPG, both of which have immune regulatory functions with OPG able to modulate T-lymphocyte responses.^50^ By changing the phenotype of circulating and tissue-resident macrophages to a restorative phenotype (F4/80^−^CD11b^+^Ly6C^+^) we have seen a reduction in liver injury.

Our data support previously published literature demonstrating that IL-10 secretion leads to a change of phenotype from circulating inflammatory monocytes to restorative macrophages.^51,52^ We have also clearly demonstrated the ability of PαS MSC to secrete IL-10 following an inflammatory stimulus, a concept that has been debated in the literature with MSC from other sources only being able to stimulate IL-10 secretion from intermediary cells, rather than secrete it themselves.^53,54^ We have demonstrated the efficacy of PαS MSC in the MDR2^−/−^ model for the first time. Of note, an ongoing area of study in the MSC literature is the ability of MSC to trigger the IMBIR,^55^ but as yet the ability of PαS MSC to trigger the IMBIR is not known and represents an area of further research. We did, however, see no indication of sudden cardiovascular collapse in any of the experiments undertaken in this study.

While no changes to overall lymphocyte numbers were seen in the MDR2^−/−^ model, we have shown that PαS MSC are able to suppress CD8^+^ proliferation and activation. This significant reduction in the CD4/CD8 ratio is in keeping with a shift from a cytotoxic to a regulatory phenotype and is biologically relevant in the setting of the pro-inflammatory state that develops following ischemia-reperfusion.

By using the MDR2^−/−^ model, we tested the effects of PαS MSC in the context of ongoing biliary injury. By doing so at an early time point in the model history, we have tested the effects on the immune-mediated component rather than the effects on fibrosis which develops later on in the time course of the model.^23^ As described by others, we have demonstrated a dose-response/threshold effect^56^ with the lower dose of MSC not significantly reducing liver injury in the MDR2^−/−^ model. Doses reported in human work vary considerably and it is difficult to compare human and mouse studies; however, the range of doses seen in clinical trials is between 0.5 × 10^5^/kg and 8.45 × 10^8^/kg.^26^ The higher dose investigated in our study represents a dose of 8 ± 1.4 × 10^6^ cells/kg, which sits comfortably within this range. While reductions in ALT and ALP were seen following MSC administration, bile acid levels remain unchanged, which is not surprising as MDR2^−/−^ mice have an ongoing biliary leak that will not be addressed by MSC therapy.

It should be noted that PαS MSC were isolated from C57BL/6 mice whereas the MDR2^−/−^ model is on an FVB background. This could be a potential limitation as some differences between strains have been demonstrated; however, given the immunological similarities between these mouse types there is no evidence to suggest that a mismatch between these two strains could explain the results seen.^57-59^ In keeping with other studies of MSC, we have demonstrated that the majority of systemically administered cells track to the lungs^49^ by using a novel cryo-imaging technique allowing single-cell resolution. In contrast to other studies, however, we have demonstrated the potential for retention/redistribution into injured liver tissue as the percentage of total MSC increased within the liver compared with other organs. This finding has only previously been demonstrated when MSC are delivered via the intraportal route.^60^ Due to the relatively low numbers of cells seen these findings may not have been detectable with lower resolution techniques. Further work to elucidate the duration by which individual cells are retained/cleared in target organs would be an interesting area of additional study as our data do not distinguish between retention and redistribution. Previous studies have demonstrated that MSC given subcutaneously do not leave the site of injection, as such this was not examined further in this study.

The route of administration of MSC is another area with conflicting results in the literature. As already discussed, there are potential advantages of direct administration to the injured area due to a large number of cells being sequestered in the lungs when administered systemically; however, this is not the whole story. Work in graft versus host disease has suggested a role for encapsulated or subcutaneous administration of MSC^36^; however, in our study, we demonstrated that in the context of liver disease, MSC delivered by this route have a negative effect that appears to be achieved by a reversal of the lymphocyte ratio to a cytotoxic phenotype. While the overall numbers of CD4^+^ and CD8^+^ did not seem to change when assessed by flow cytometry the balance between them did. It is known that MSC are able to modify levels of CD4^+^ and CD8^+^ cells and perhaps the assessment of the ratio of these cells is capturing a subtle change not seen in the total numbers.

The differences between systemic and subcutaneous administration also raise concerns, particularly as the subcutaneous route appeared to increase the liver injury seen. The mechanism behind these differences is not immediately clear, although it may represent a difference between direct cell contact and the effects of MSC secreted factors. While cell-to-cell contact has been shown to represent a mechanism by which MSC can suppress lymphocyte proliferation and activation,^56^ more recent studies have focused on factors secreted by MSC rather than the MSC themselves.^61^ This study suggests that the truth may be more complex and that route of administration may need to be considered along with other factors. These findings should act as a caution when designing translational studies as MSC have the ability to worsen disease as well as improve it. This difference between our study and other published work may be explained by differences between systemic and more organ-specific disease processes with MSC exerting a different effect when directly exposed to areas of injury.

Ischemia-reperfusion models are accompanied by extensive immune-mediated damage, and so as to maximize the potential benefit of MSC therapy in this setting we used doses at the higher end of those reported in the published literature. With this in mind, and given concerns over high doses of MSC triggering acute thromboembolic phenomena when given systemically we determined that the intraperitoneal route of administration was the best from a risk-benefit ratio. Subcutaneous administration in the hepatic ischemia-reperfusion model was discounted based on previous negative studies in this model already published.^62,63^ A significant proportion of the acute damage seen in liver transplantation occurs during the reperfusion phase. With this in mind, we tested PαS MSC in a surgical model of hepatic ischemia-reperfusion injury. While we did not show a beneficial effect when using markers of hepatocyte injury such as ALT, we did show a reduction in cell death using PAS staining as a surrogate marker of cell death commonly used in clinical practice. While there have been reports of MSC therapy reducing ALT in other studies, these are based on rat models of ischemia-reperfusion injury^39,64^ and as such are not directly comparable due to the inherent differences in the rat, mouse, and human immune systems, with mice showing a greater similarity to humans with regards to their immune system function.

In order to translate the findings in this preclinical study into a clinical environment, further work will need to be performed. While PαS MSC are a murine MSC there are similar human analogs.^65^ This study suggests that in the context of liver injury, systemic administration may be favorable when compared with subcutaneous routes without causing an increase in adverse events; however, this would need to be considered in the context of other reports of thromboembolic complications,^33^ and while the evidence for thromboembolic complications so far is limited, given the significant consequences careful consideration needs to be taken when administering MSC in clinical trials.^35^ This study also proposes a benefit of a less heterogeneous population MSC isolated on specific markers and trialing such cells in the clinical environment may confer a benefit over more conventional cell therapy products. Translating murine cell therapy doses to human studies can be challenging, particularly if using different cells. In this study the doses were in the middle of ranges commonly seen in clinical trials, ±0.7 × 10^6^ cells/kg and 8 ± 1.4 × 10^6^ cells/kg, representing a suitable starting point for a translational study, minimizing the risks while maximizing the potential for a therapeutic effect.^26^

## Conclusions

In this study of purified murine MSC, we have demonstrated a clear improvement in liver injury and inflammation in models representative of that seen following a DCD transplantation through their ability to secrete IL-10 and OPG and switch macrophage and lymphocyte phenotypes to a restorative type. We have also suggested a role in reperfusion injury. These findings suggest a potential role for MSC in marginal organ transplantation that merits further study.

## Supplementary Material

sxac059_suppl_Supplementary_Figure_1

## Data Availability

The data underlying this article will be shared on reasonable request to the corresponding author.
