# Supplementary figures and images for: PDGFRα/Sca-1 Sorted Mesenchymal Stromal Cells Reduce Liver Injury in Murine Models of Hepatic Ischemia-Reperfusion Injury
Source: Stem Cells. 2022 Aug 24;40(11):1056–70. doi: 10.1093/stmcls/sxac059 (PMC9707286; doi:10.1093/stmcls/sxac059)

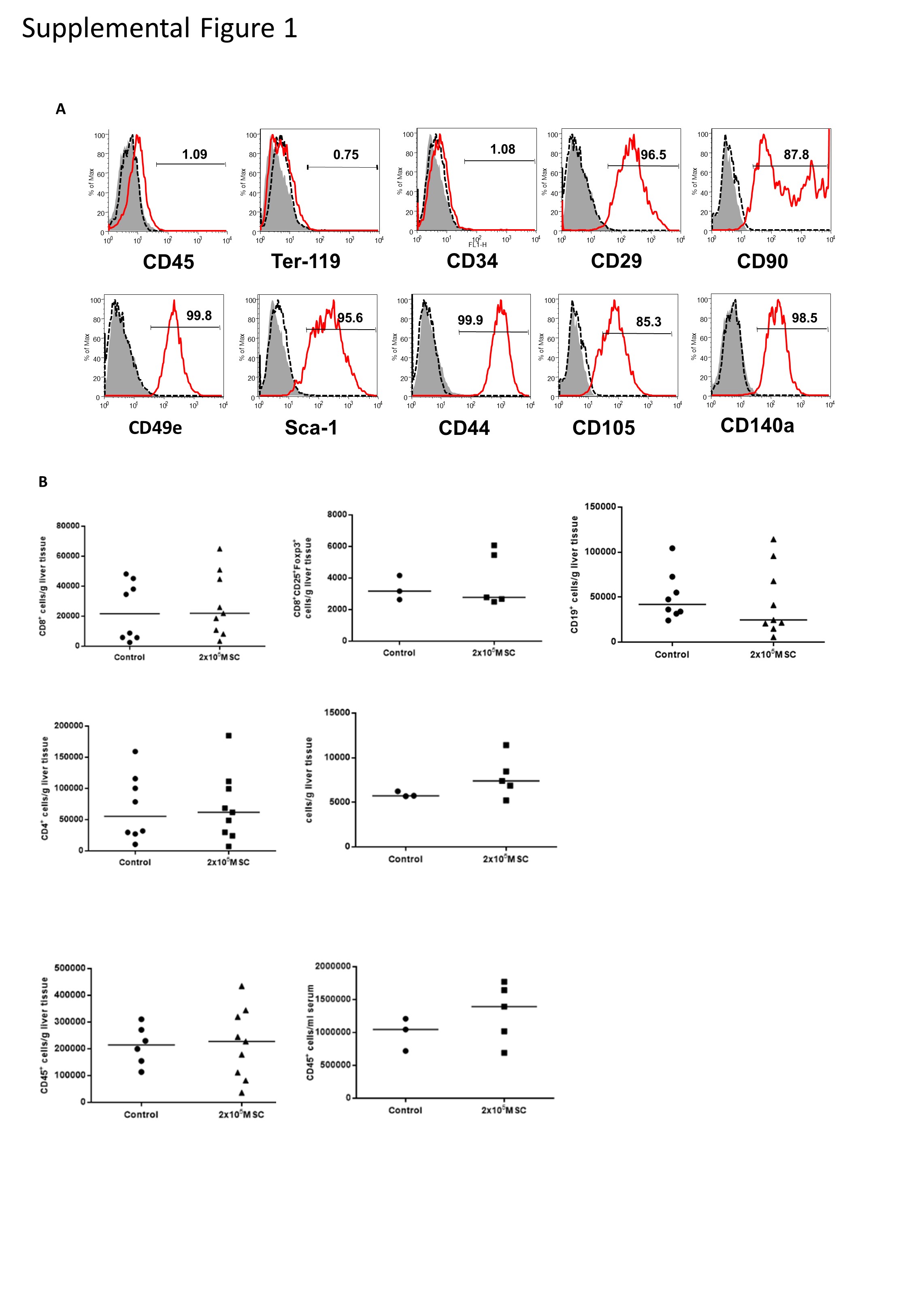

Supplement: sxac059_suppl_Supplementary_Figure_1 [file sxac059_suppl_supplementary_figure_1.jpeg]
